# Negative regulation of floral transition in Arabidopsis by HOS15-PWR-HDA9 complex

**DOI:** 10.3389/fpls.2022.1105988

**Published:** 2023-01-06

**Authors:** Chae Jin Lim, Ki Suk Park, Akhtar Ali, Junghoon Park, Seung Min Ryou, Mingzhe Shen, Haris Ali Khan, Zein Eddin Bader, Shah Zareen, Min Jae Bae, Jong Hyoo Choi, Zheng-Yi Xu, Jose M. Pardo, Dae-Jin Yun

**Affiliations:** ^1^ Institute of Global Disease Control, Konkuk University, Seoul, Republic of Korea; ^2^ Department of Biomedical Science and Engineering, Konkuk University, Seoul, Republic of Korea; ^3^ Department of Agronomy, Agricultural College, Yanbian University, Yanji, China; ^4^ Key Laboratory of Molecular Epigenetics of the Ministry of Education (MOE), Northeast Normal University, Changchun, China; ^5^ Institute of Plant Biochemistry and Photosynthesis, Consejo Superior de Investigaciones 17 Cientificas and Universidad de Sevilla, Seville, Spain

**Keywords:** Arabidopsis, flowering time, chromatin remodeling, histone acetylation and deacetylation, HOS15, HDA9, PWR, AGL19

## Abstract

Arabidopsis HOS15/PWR/HDA9 repressor complex, which is similar to the TBL1/NcoR1/HDAC complex in animals, plays a well-known role in epigenetic regulation. PWR and HDA9 have been reported to interact with each other and modulate the flowering time by repressing *AGL19* expression, whereas HOS15 and HDA9, together with the photoperiodic evening complex, regulate flowering time through repression of GI transcription. However, the role of the HOS15/PWR/HDA9 core repressor complex as a functional unit in the regulation of flowering time is yet to be explored. In this study, we reported that the loss-of-function *hos15-2/pwr/hda9* triple mutant accumulates higher transcript levels of *AGL19* and exhibits an early flowering phenotype similar to those of *hos15*, *pwr*, and *hda9* single mutants. Interestingly, the accumulation of HOS15 in the nucleus was drastically reduced in *pwr* and *hda9* mutants. As a result, HOS15 could not perform its role in histone deacetylation or interaction with H3 in the nucleus. Furthermore, HOS15 is also associated with the same region of the *AGL19* promoter known for PWR-HDA9 binding. The acetylation level of the *AGL19* promoter was increased in the *hos15-2* mutant, similar to the *pwr* and *hda9* mutants. Therefore, our findings reveal that the HOS15/PWR/HDA9 repressor complex deacetylates the promoter region of *AGL19*, thereby negatively regulating *AGL19* transcription, which leads to early flowering in Arabidopsis.

## Introduction

The regulation of flowering time has been well studied in Arabidopsis and involves three major pathways: vernalization, autonomous, and photoperiod pathways. Exposure to cold temperatures in winter is necessary for flowering in spring or summer, and this process is called vernalization (Amasino, 2005). The autonomous or developmental pathway is established by repressing FLOWERING LOCUS C (FLC), a negative regulator of floral integrators, thereby activating the expression of flowering genes, including the mobile ‘florigen’ FLOWERING LOCUS T (FT) (Corbesier and Coupland, 2006). FLC abundance is also repressed by vernalization independently of the autonomous pathway. Lastly, the photoperiod pathway is controlled by light and the circadian clock. The major factors involved in the photoperiod pathway are CONSTANS (CO) and GIGANTEA (GI), that integrate clock and light signals to provide photoperiod-specific induction of *FT* expression (Jarillo et al., 2008; Song et al., 2014; Shim et al., 2017).

AGAMOUS LIKE 19 (AGL19) was first discovered through phylogenetical identification of conserved MADS-box domain (Alvarez-Buylla et al., 2000). Expression of *AGL19* is mainly expressed in roots and is also observed in leaves, seedlings, and flowers. The first function of *AGL19* was revealed as a floral activator in the vernalization pathway. AGL19 in this pathway is negatively regulated by POLYCOMB REPRESSIVE COMPLEX 2 (PRC2) subunits and these complexes also target *AGL19* chromatin region and affect H3K27me3 enrichment (Schönrock et al., 2006). *AGL19* functions not only in flowering related to vernalization but also in non-inductive short-day conditions. To prevent precocious flowering, de-acetylation activity of HISTONE DEACETYLASE 9 (HDA9) regulates the acetylation level of *AGL19*, which in turn results in repression of *FT* gene (Kang et al., 2015).

Gene regulation in signaling pathways is determined genetically and epigenetically. Epigenetic regulation of genes is achieved by histone modification, chromatin remodeling, DNA methylation, non-coding RNA, and microRNA (Chinnusamy and Zhu, 2009). Histone modification is a well-known factor involved in epigenetic gene regulation. Histones are modified by acetylation, phosphorylation, ubiquitination, methylation, and sumoylation, which alter the structure of chromatin by either compacting or loosening it (Pfluger and Wagner, 2007; Alaskhar Alhamwe et al., 2018; Zhao et al., 2019). Histone acetylation relaxes chromatin, making it possible for transcription factors to bind to the promoters, thereby activating gene expression. On the contrary, chromatin becomes compacted with histone deacetylation, hindering the interaction of transcription factors with the promoters and repressing the expression of genes (Tamaru, 2010; Park et al., 2018a).

Acetylation and deacetylation of histones are mediated mainly by histone acetyltransferases (HATs) and histone deacetylases (HDACs), respectively. In plants, HDACs are classified into three groups: the RPD3/HDA1 superfamily, HD-tuin family, and SRT (sirtuin) family (Pandey et al., 2002; Hollender and Liu, 2008). Plant HATs are also classified into three groups: GNAT (GCN5- RELATED N-TERMINAL ACETYLTRANSFERASE)-MYST, p300/CREB binding protein (CBP), and TAF_II_250 related (Pandey et al., 2002). HDACs and HATs in plants regulate numerous plant genes, including those involved in plant development, flowering, and stress responses. For instance, HDA9, an RPD3/HDA1 type histone deacetylase, regulates floral transition in Arabidopsis. HDA9 negatively regulates the flowering-inducer gene AGAMOUS-LIKE 19 (*AGL19*) by deacetylating its promoter region (Kim et al., 2013; Kim et al., 2016). HDA9 also interacts with the promoters of the flowering genes *SOC1* and *AGL24* in *Brassica juncea* (Jiang et al., 2018). In addition, HDA9 is a major component of the evening complex that represses GI (Park et al., 2019).

The Arabidopsis protein HIGH EXPRESSION OF OSMOTICALLY RESPONSIVE GENES 15 (HOS15) is a homolog of mammalian TBL1 and TBLR1, which are core components of the SMRT/NCoR1 co-repressor complex (Perissi et al., 2008; Park et al., 2018b). Further analysis revealed that HOS15 is also important for histone deacetylation, supporting the notion that HOS15 functions as a repressor of gene expression (Zhu et al., 2008). Structural analysis showed that HOS15 comprises four major motifs: the LisH motif, WD-40 domain, F-box-like motif, and DWD motif. The LisH motif is involved in protein dimerization (Choi et al., 2008; Joachimiak et al., 2020). The WD-40 motif is important for protein-protein interactions (Xu and Min, 2011). The F-box and DWD motifs are well-known domains for the substrate receptor in the Cullin-base E3 ubiquitin ligase complex. Recent findings suggest that HOS15 acts as a ubiquitin E3 ligase substrate receptor to regulate various signal transduction pathways through targeted protein degradation (Park et al., 2018b; Ali et al., 2019; Shen et al., 2020). HOS15 promotes the degradation of HD2C in low-temperature signaling, regulates drought stress through OST1 (SnRK2.6) degradation, and is responsible for immune responses by targeting NPR1 for degradation (Park et al., 2018b; Ali et al., 2019; Shen et al., 2020). More recently we have shown that HOS15 interacts with POWERDRESS (PWR), known as the SMRT/NCoR1 homolog, and regulates the stability of the HD2C protein under cold stress (Lim et al., 2020). Moreover, it was recently reported that HOS15 in conjunction with PWR-HDA9 regulates leaf senescence (Zareen et al., 2022). HDA9 also forms a complex with PWR and acts as a repressor in various signaling pathways, such as flowering time, leaf senescence, development, thermomorphogenesis, ABA and drought stress response (Yumul et al., 2013; Kang et al., 2015; Chen et al., 2016; Kim et al., 2016; Suzuki et al., 2018; Tasset et al., 2018; Mayer et al., 2019; Ali and Yun, 2020; Khan et al., 2020; Zareen et al., 2022). Overall, these reports suggest the existence of a HOS15-PWR-HDA9 repressor complex which plays an important role in the overall growth, development, and leaf senescence of plants, as well as in overcoming external stimuli.

HOS15 and HDA9 have been shown to be important components of the evening complex that regulate *GI* (Park et al., 2019). In a similar study, the PWR-HDA9 complex was shown to negatively regulate the expression of the *AGL19* through histone deacetylation (Kang et al., 2015; Kim et al., 2016). However, the molecular basis of HOS15-PWR-HDA9 complex interference with flowering time is yet to be determined. In this study, we report that HOS15, together with PWR and HDA9, forms a repressor complex and negatively regulates floral transition by inhibiting the expression of *AGL19* through histone deacetylation. Furthermore, HDA9 and PWR are required for the nuclear translocation of HOS15 to repress the expression of target genes. Plants lacking the HOS15-HDA9-PWR complex exhibit hyperacetylation of the *AGL19* promoter region, which leads to an early flowering phenotype. Our findings demonstrated that HOS15-HDA9-PWR is a repressor complex that negatively regulates floral transition through histone deacetylation.

## Materials and methods

### Plant materials and growth conditions

The *Arabidopsis thaliana* ecotype Col-0 and mutants *pwr*-2 (SALK_071811), *pwr*-3 (SALK_006823), *hda9-1* (GK_305G03), *hda9-2* (SALK_007123), and *hos15-2* (GK_785B10) mutants, and *HOS15pro::HOS15-HA/hos15-2* (Park et al., 2018a), *HOS15pro::HOS15-FLAG/hos15-2* (Park et al., 2018b) transgenic plants were used in this study. Double (*pwr-2 hos15-2*, *hda9-2 hos15-2*, *pwr-2 hda9-2*) and triple mutants (*pwr-2 hda9-2 hos15-2*) were generated by crossing. Seeds were surface-sterilized in a solution containing 3% (v/v) sodium hypochlorite solution (Yakuri Pure Chemicals, Kyoto, Japan) for 5 min and then rinsed five times with sterilized water. After stratification at 4°C for 3 days in the dark, the plants were grown on half-strength Murashinge and Skoog (MS) medium or soil at 23°C ± 1°C under 16-h light/8-h-dark conditions with 100 µM m^-2^s^-1^ light intensity.

### Flowering time measurements

Flowering time analysis of the WT and mutant plants was performed at 23°C ± 1°C in long-day conditions. Flowering time was measured by counting the number of days-to-flowering and the total number of rosette leaves from the primary meristems at bolting.

### RNA extraction and quantitative PCR analysis

Total RNA was extracted from two-week-old plants using the RNeasy Plant Mini Kit (Qiagen) and treated with DNase (Sigma-Aldrich). cDNA was synthesized using reverse transcriptase (Toyobo, Osaka, Japan). The SYBR Green PCR Master Mix Kit (Bio-Rad) was used for quantitative PCR using the indicated gene-specific primers. Primer sequences are provided in **Supplementary Table 1**. Relative expression levels were analyzed using the comparative cycle threshold method (Bio-Rad).

### Protein extraction and western blot analysis

Total protein was isolated from Arabidopsis seedlings using protein extraction buffer containing 150 mM NaCl, 100 mM Tris-Cl (pH 7.5), 0.5% (v/v) NP-40, 1 mM EDTA, 3 mM DTT, and protease inhibitors (1 mM PMSF, 5 μg mL^-1^ leupeptin, 1 μg mL^-1^ aprotinin, 1 μg mL^-1^ pepstatin, 5 μg mL^-1^ antipain, 5 μg mL^-1^ chymostatin, 2 mM Na_2_VO_3_, 2 mM NaF, and 50 mM MG132). The proteins were separated and detected using SDS-PAGE and immunoblottied with the appropriate antibodies, anti-HOS15 (Park et al., 2018a; Lim et al., 2020), anti-α-tubulin (Sigma-Aldrich), anti-Ac-H3 (Millipore), anti-H3 (Abcam), and anti-FLAG (Sigma-Aldrich) for 2 h at room temperature or overnight at 4°C. Membranes were developed using peroxidase-conjugated secondary anti-rabbit IgG (Santa Cruz Biotechnology) and anti-mouse IgG (Santa Cruz Biotechnology). Western blot membranes were incubated with Clarity™ Western ECL Substrate solution (Bio-Rad), and signals were detected using an imaging system (ChemiDoc™MP, Bio-Rad).

### Nuclear-cytoplasmic fractionation assay

Nuclei were extracted from two-week-old plants using Honda’s buffer (0.4 M sucrose, 2.5% Ficoll 400, 5% dextran T-40, 10 mM MgCl_2_, 25 mM Tris-Cl (pH 7.5), 10 mM β-mercaptoethanol, 100 mg/mL phenylmethylsulfonyl fluoride, 0.5 mg/mL antipain, and 0.5 mg/mL leupeptin) (Folta and Kaufman, 2006; Park et al., 2018b). The samples were filtered through a 60-µm nylon filter (Millipore) and incubated on ice for 15 min. Triton X-100 was added at a final concentration of 0.5% and the sample was centrifuged at 1500*g* for 5 min. The supernatants were transferred to new tubes, and cytosolic proteins were used. The pellet was washed with 1 mL Honda’s buffer containing 0.1% Triton X-100. The cells were gently resuspended and centrifuged at 100*g* for 5 min to pellet the starch and cell debris. The supernatant was transferred to a tube and centrifuged at 1800*g* for 5 min to pellet the nuclei. The nuclear and cytosolic extracts were suspended in SDS-PAGE loading buffer. Immunoblots were performed using anti-HOS15, anti-α-tubulin (Sigma), and anti-H3 (Abcam) antibodies, and antigen proteins were visualized by chemiluminescence using ECL-detecting reagent (Bio-Rad).

### ChIP assay

The chromatin immunoprecipitation (ChIP) assay was performed as described by Lim et al. (2020). Nuclear proteins were extracted from two-week-old Arabidopsis plants, and immunoprecipitation was performed using salmon sperm DNA/protein A agarose beads (Upstate Biotechnology) fused to anti-HOS15 and anti-acetylated H3 antibodies (Millipore). Beads treated with anti-rabbit IgG were used as negative controls for each ChIP assay. The immunoprecipitated DNA was purified by phenol-chloroform-isoamyl (PCI) extraction and ethanol precipitation. The precipitated DNA was dissolved in TE buffer and quantified using RT-qPCR. The primers used in the ChIP assays are listed in **Supplementary Table 1**, and an *UBQ10* DNA fragment was used for normalization.

## Results

### Negative regulation of flowering time by HOS15-PWR-HDA9 *via* inhibition of *AGL19* transcription

To investigate the function of the HOS15-PWR-HDA9 complex in regulating the flowering time, we analyzed flowering phenotypes of the loss-of-function *hos15-2* mutant and three complementation lines expressing the construct *HOS15pro::HOS15-HA* in the *hos15*-*2* background (Park et al., 2018a). As expected, *hos15-2* mutant plants exhibited early flowering (Park et al., 2019), while the flowering time of complementation lines was similar to that of WT plants (**Supplemental Figures 1A–C**). Using RNA-seq analysis data, we previously showed that the expression level of the *AGL19* was upregulated in *hos15-2* mutants (Park et al., 2019), which could promote flowering time (Kang et al., 2015; Park et al., 2019). To confirm this, we analyzed the transcript level of *AGL19* using RT-qPCR, which was significantly increased in the *hos15-2* mutant. In contrast, the complementation lines possessed a similar level of *AGL19* transcription to that of the WT plants (**Supplemental Figure 1D**; **Supplemental Figure 2**). These results suggest that the early flowering phenotype of *hos15-2* might be due to the upregulation of *AGL19.*

**Figure 1:**
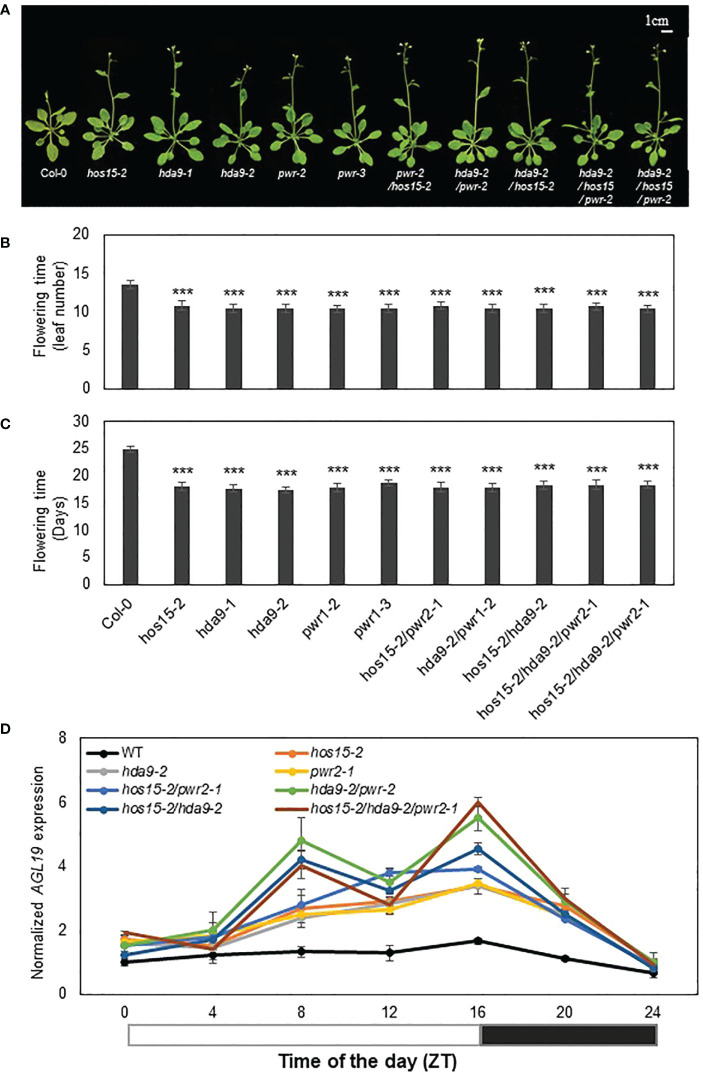
Involvement of HOS15, HDA9, and PWR in the same flowering pathway. **(A–C)** Flowering time of wild type (Col-0), *hos15-2*, *hda9*, *pwr-2*, as well as double and triple mutant plants under long-day conditions (16 h light/8 h dark). The number of rosette leaves at bolting **(B)** and days to flowering **(C)** were counted from **(A)**. Data represent means ± SD (n = 19). Significant difference was determined by a Student’s t-test (***p<0.001). **(D)** The transcript level of *AGL19* in two-week-old Col-0, *hos15-2*, *hda9*, *pwr*, as well as double and triple mutant Arabidopsis plants. Samples were harvested according to Zeitgeber time (ZT) and were analyzed by RT-qPCR and normalized with ACTIN2. Data represent means ± SD from three biological replicates with three technical repeats each (n = 3). The black and white bar represents night and daylight periods, respectively.

The transcript level of *AGL19* has previously been reported to be upregulated in *pwr* and *hda9* knockout mutants, and as a result, these mutants show early flowering phenotypes (Kang et al., 2015; Kim et al., 2016). Since HOS15 knockout plants also show early flowering and the upregulation of *AGL19* transcript levels (**Supplemental Figure 1**), we assumed that HOS15 might work together with PWR-HDA9 in the same repressor complex, thereby negatively regulating flowering time by repressing *AGL19* transcription. To test our hypothesis, we generated double and triple (*pwr hos15*, *hda9 pwr, hda9 hos15, hda9 hos15 pwr*) mutant plants and tested their flowering phenotypes. As expected, double and triple knockout mutants of the HOS15-PWR-HAD9 complex showed an early flowering phenotype statistically similar to single mutants of HOS15, PWR, and HDA9, signifying that these mutations were not additive (**Figure 1A–C**). In addition to flowering phenotypes, the transcript level of *AGL19* was upregulated in single, double, and triple mutant plants compared to that in WT plants (**Figure 1D**). Furthermore, double and triple mutants of HOS15, PWR, and HDA9 exhibited dwarf phenotypes, such as small plant size and short and blunt-end siliques, which are common phenotypes of *hos15-2*, *pwr*, and *hda9* single mutants (**Supplemental Figures 3 and 4**) (Kang et al., 2015; Kim et al., 2016; Mayer et al., 2019; Lim et al., 2020). Overall, these findings indicate that the HOS15-PWR-HDA9-complex negatively regulates flowering time by inhibiting *AGL19* expression.

**Figure 2:**
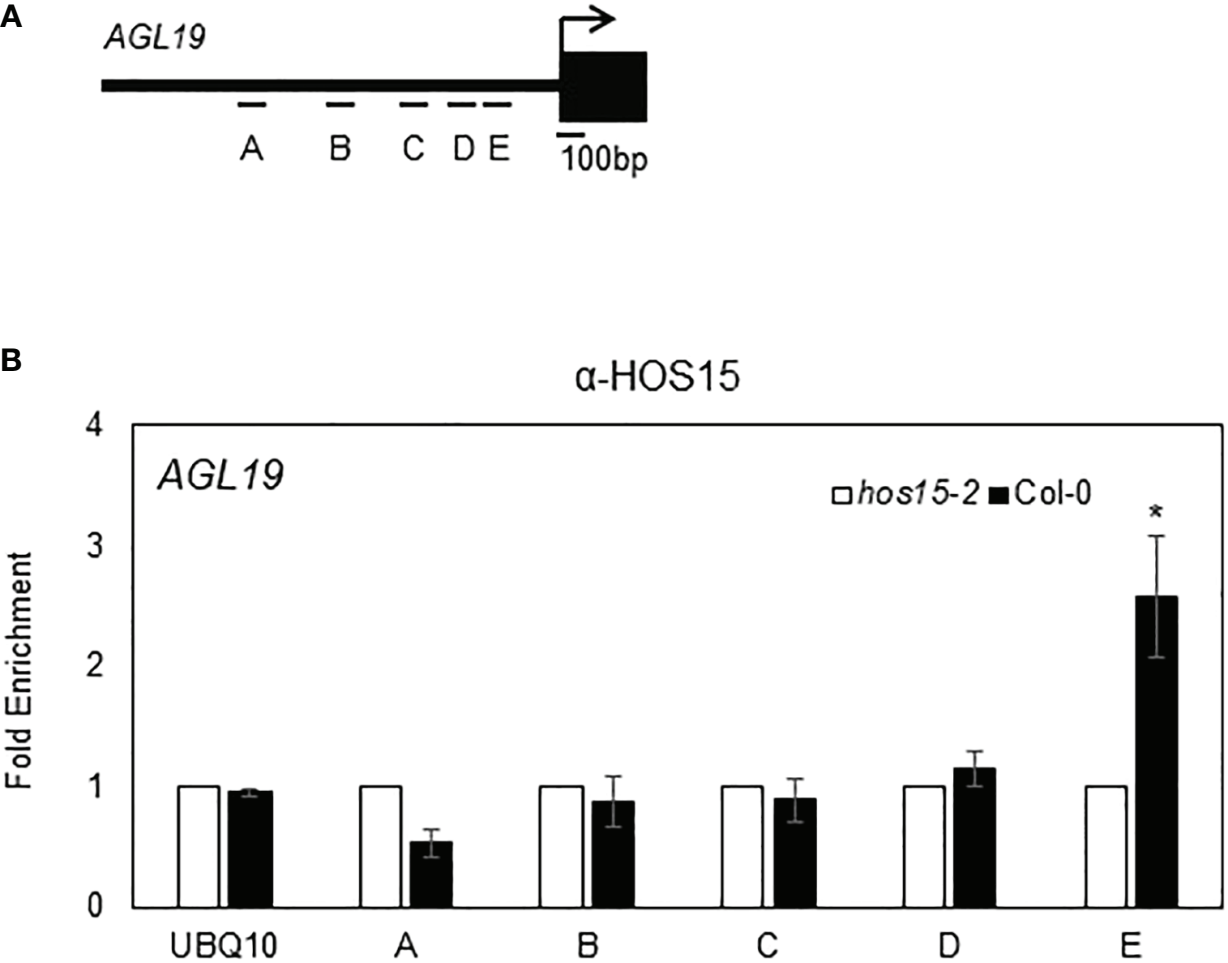
Association of HOS15 with the *AGL19* promoter region. **(A)** Amplicon regions (A–E) in the *AGL19* promoter used for ChIP-qPCR. The arrow indicates the transcriptional start site. **(B)** The ChIP assays were performed using the anti-HOS15 antibody on samples from two-week-old wild-type (Col-0) and *hos15-2* mutant plants. The amount of DNA in the immunoprecipitated complex was determined by RT-qPCR using primers specific to the different regions (A–E) of the *AGL19* promoter and is presented as the fold enrichment after normalization with the corresponding input and compared with *hos15-2* mutant plants. Data represent means ± SD from three biological replicates with three technical repeats each (n = 3). Significant difference was determined by a Student’s t-test (* p<0.05).

HDA9 negatively regulates *AGL19* expression by associating with the promoter region of *AGL19* and promoting histone deacetylation (Kang et al., 2015). Therefore, we tested whether HOS15 is associated with the promoter region of *AGL19* to modulate chromatin status. A chromatin immunoprecipitation (ChIP) assay showed that HOS15 was indeed associated with the E region of the *AGL19* promoter, in a region already known for HDA9 association (**Figure 2**; Kang et al., 2015). These data suggest that HOS15 binds to the promoter of *AGL19* to co-regulate the histone acetylation status of *AGL19* chromatin.

### Role of PWR-HDA9 in HOS15 nuclear accumulation

**Figure 3:**
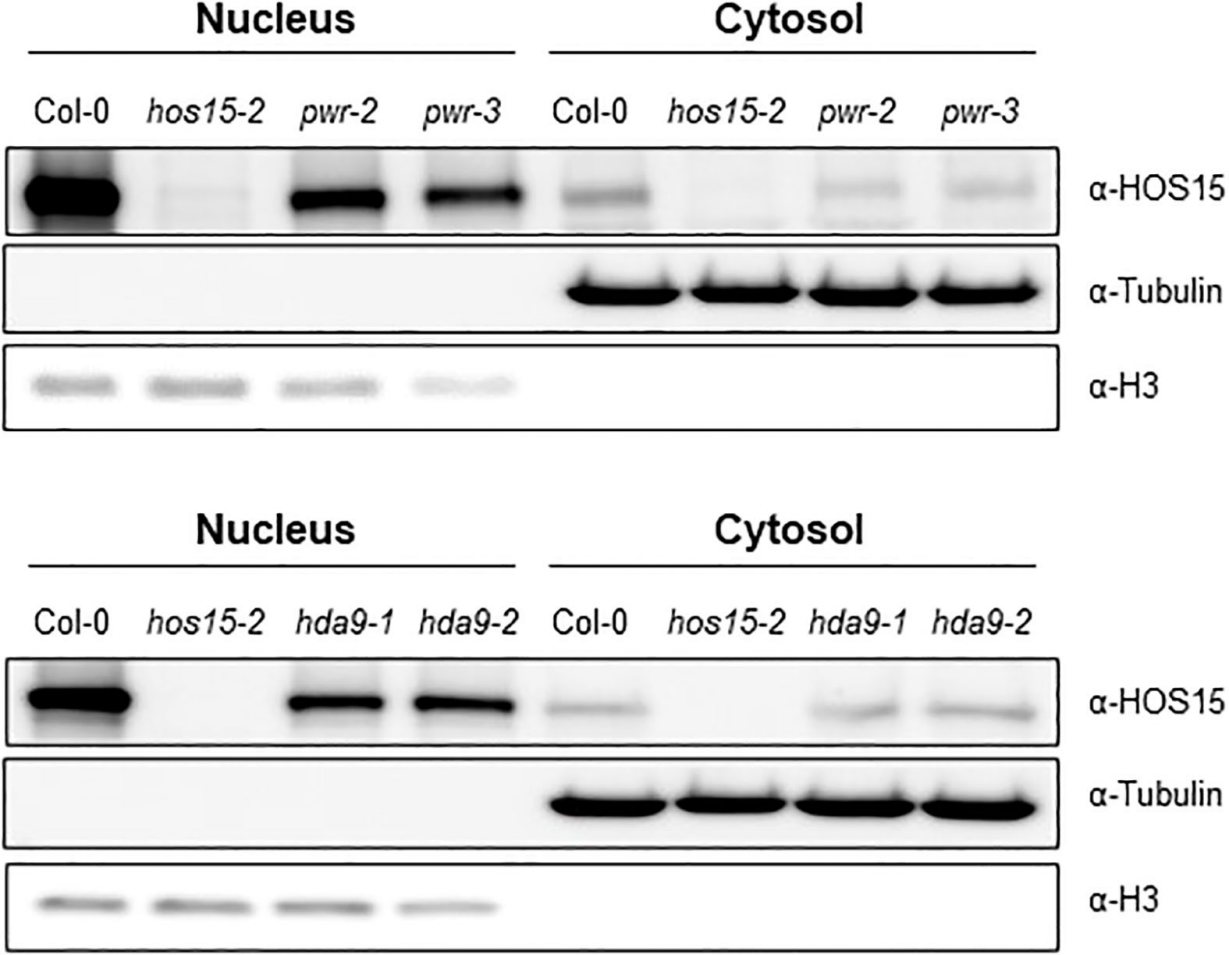
Decreased HOS15 nuclear accumulation in *pwr* and *hda9* mutants. Immunoblotting of HOS15 protein in two-week-old plants of Col-0, *hos15-2*, *hda9* and *pwr* mutants. Total protein was extracted and separated into cytoplasmic (cytosol) and nuclear (nucleus) fractions and immunoblotted with an anti-HOS15 antibody. α-Tubulin and histone H3 served as loading controls for the cytosol and nucleus, respectively.

HOS15 forms a complex with PWR and HDA9 (Park et al., 2018b; Suzuki et al., 2018; Mayer et al., 2019). Furthermore, HOS15, PWR, and HDA9 were predominantly localized in the nucleus (Zhu et al., 2008; Kang et al., 2015; Lim et al., 2020), and HDA9 accumulation was reduced in the nuclei of *pwr* and *hos15* mutant plants (Chen et al., 2016; Mayer et al., 2019). Based on these precedents, we investigated whether the accumulation of HOS15 in nuclei also required PWR-HDA9. A nuclear-cytoplasm fractionation analysis using *pwr* and *hda9* mutant plants showed that HOS15 nuclear accumulation was greatly decreased in *pwr* and *hda9* mutants as compared with WT (**Figure 3**). Notably, compared with WT plants, *HOS15* transcript levels were not changed in *pwr* and *hda9* mutants; in contrast, the total protein amount was decreased in these mutants (**Supplemental Figure 5**). HOS15 also interacts with other RPD3-type class 1 histone deacetylases, such as HDA6 and HDA19 (Park et al., 2018a; Park et al., 2018b). However, unlike *hda9*, *hda6*, and *hda19* mutants did not exhibit early flowering; instead, they exhibited partial late-flowering phenotypes (**Supplemental Figures 6A, B**). Furthermore, HOS15 total protein remained unchanged in *hda6* and *hda19* mutant plants compared with that in WT plants (**Supplemental Figure 6C**). Overall, these results suggest that HOS15 forms a complex with PWR-HDA9 in the nucleus and regulates floral transition by repressing *AGL19* expression.

### Interaction of HOS15 with H3 and its regulation of histone deacetylation

**Figure 4:**
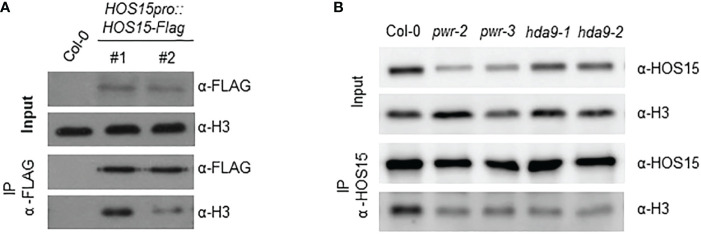
Interaction of HOS15 with histone H3. **(A)** Co-IP assay using two-week-old Arabidopsis Col-0 and *HOS15pro:HOS15-Flag/hos15-2* complementation lines. Protein extracts (Input) were immunoprecipitated with anti-FLAG antibody and immunoblotted with anti-FLAG and anti-H3 antibodies **(B)** Co-IP assay using two-week-old Arabidopsis Col-0 *hos15-2*, *hda9* and *pwr* mutant plants. Protein extracts (Input) were immunoprecipitated with anti-HOS15 antibody and immunoblotted with anti-HOS15 and anti-H3 antibodies.

**Figure 5:**
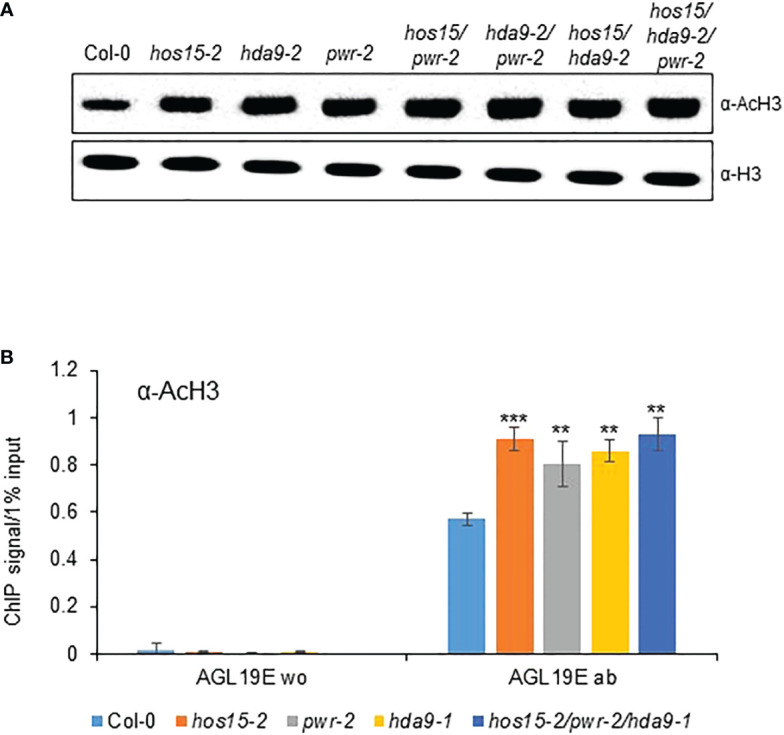
Regulation of *AGL19* histone acetylation by PWR-HOS15-HDA9 complex. **(A)** Total proteins were extracted from two-week-old Col-0, *hos15-2*, *hda9*, *pwr*, as well as double and triple mutant plants and immunoblotted with anti-AcH3 antibody. Antibodies against histone H3 were used as the loading control. **(B)** Chromatin complexes from Col-0, *hos15, pwr-2, hda9-1*, and *hos15-2 pwr-2 hda9-1* triple mutant plants were immunoprecipitated with anti-AcH3 (*ab*). A control reaction was processed in parallel with rabbit IgG only (*w/o*). ChIP and input-DNA samples were quantified by real-time qPCR using primers specific to the different regions **(A–E)** of the *AGL19* promoter region. Data represent means ± SD from three biological replicates with three technical repeats each (n = 3). Significant difference was determined by a Student’s t-test (**p<0.01, ***p<0.01).

Previous reports have revealed that HOS15, PWR, and HDA9 repress target gene expression by regulating histone H3 acetylation status (Kim et al., 2016; Mayer et al., 2019; Park et al., 2019). To investigate whether HOS15 interacts with H3, we performed a co-IP assay using two independent *HOS15pro::HOS15-Flag*/*hos*15-2 complementation plant lines (Park et al., 2018b). As shown in **Figure 4A**, HOS15 interacted with H3 *in vivo*. As HOS15 stability in the nucleus is dependent on PWR-HDA9, we tested HOS15 and H3 interactions in *pwr* and *hda*9 mutants. Notably, the interaction of HOS15 with H3 was drastically reduced in *pwr* and *hda9* mutants compared with that in the WT plants (**Figure 4B**). In addition, total histone acetylation levels were increased in *hos15*, *pwr* and *hda9* single, double, and triple mutant plants compared with WT plants, and the increment was similar between single and higher order mutants (**Figure 5A**) (Zareen et al., 2022). In line with this, we next tested the acetylation status of *AGL19* chromatin in *hos15*, *pwr* and *hda9* single and *hos15/pwr/hda9* triple mutant plants using a ChIP assay with acetylated H3 (AcH3) antibody. H3 acetylation of *AGL19* chromatin was higher in *hos15-2* mutant plants than in the WT plants (**Figure 5B**, **Supplemental Figure 7**). Consistent with the notion that HOS15 functions together with the PWR-HDA9 complex, no significant difference in the increase in H3 acetylation level was observed between these single and triple mutant plants (**Figure 5B**). Taken together, these findings suggest that HOS15, PWR, and HDA9 work together in the same repressor complex and deacetylate *AGL19* chromatin region, thereby negatively regulating *AGL19* expression.

## Discussion

### Epigenetic regulation of flowering time in Arabidopsis by HOS15-PWR-HDA9 co-repressor complex

**Figure 6:**
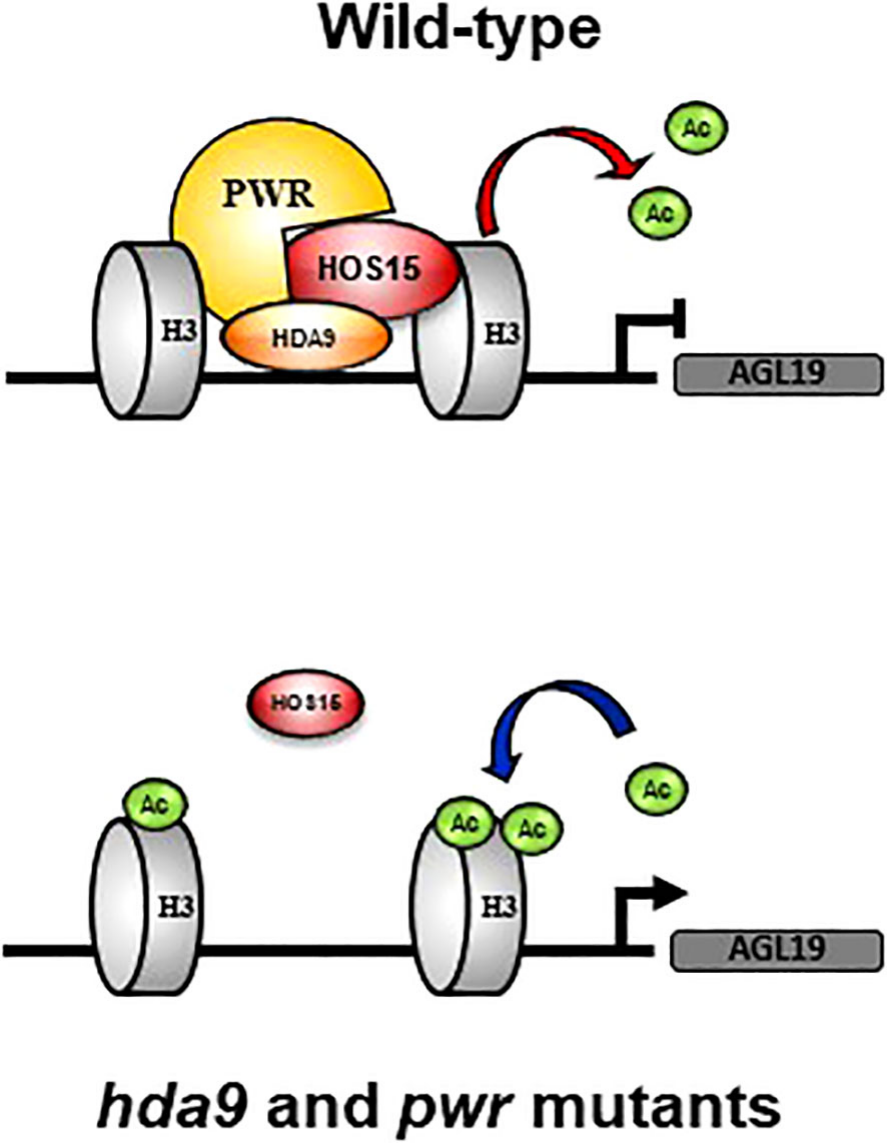
Working model for HOS15-PWR-HDA9 complex in flowering. Under natural conditions (wild-type), the HOS15-PWR-HDA9 complex negatively regulates *AGL19* gene expression through histone deacetylation. In the absence of PWR and HDA9, which interact with HOS15 to form a complex, the accumulation of HOS15 in the nucleus is reduced. Disruption of the HOS15-PWR-HDA9 repressor complex results in enhanced histone acetylation and relaxed chromatin. *AGL19* gene expression increases compared to that in the wild type, and flowering is promoted.

Epigenetic regulation plays an essential role in various signaling pathways in plants. Our study revealed the role of the HOS15-PWR-HDA9 co-repressor complex in the epigenetic regulation of flowering time through the expression of *AGL19*, a flowering-regulating gene (Kim et al., 2013; Kang et al., 2015). Although the regulatory pathways leading to flowering in Arabidopsis have been well documented, there is still room for further investigation to uncover the complete mechanisms of floral transitions. Using genetic interactions, proteomics, and chromatin immunoprecipitation assays, we found that the HOS15-PWR-HDA9 co-repressor complex inhibits *AGL19* expression through histone modification of *AGL19* chromatin thereby negatively regulating flowering time in Arabidopsis (**Figure 6**). Loss-of-function single, double, or triple mutants of the HOS15-HDA9-PWR complex resulted in early flowering phenotypes (**Figure 1**). Furthermore, the expression of *AGL19* was strongly upregulated in *hos15*, *hda9* and *pwr* mutants, which highlights the involvement of HOS15-HDA9-PWR complex in floral transition. The diurnal expression pattern of *AGL19* was altered in the triple and the double mutants *hos15 hda9* and *pwr hda9* with a decline at ZT12 (**Figure 1**). The reason for this behavior is unknown, but the HOS15/PWR/HDA9 complex is likely to impact the expression of other circadian and photoperiodic genes whose altered expression may, in turn, impinge on the diurnal rhythm of *AGL19* transcripts enhancing the oscillations (Brambilla and Fornara, 2017).

### HDA9 and PWR are required for HOS15’s function in the nucleus

Previously we have shown that HDA9 and HOS15 regulate flowering time by interfering with GI transcription (Park et al., 2019). We have also shown that PWR is required for HOS15-mediated HD2C degradation upon cold stress (Lim et al., 2020). These findings suggest that HDA9 and PWR are required for functional HOS15. To confirm this, we analyzed HOS15 accumulation in *hda9* and *pwr* mutants both in nucleus and cytosol. As shown in **Figure 3**, HOS15 accumulation in the nucleus was greatly reduced in *hda9* and *pwr* mutants compared with WT. By contrast, HOS15 accumulation in the cytosol was almost similar among all tested lines (**Figure 3**), suggesting that HDA9 and PWR are required for nuclear accumulation of HOS15. Furthermore, HOS15 interaction with H3 was impaired in *hda9* and *pwr* mutants (**Figure 4**) which further strengthened the notion that functional HDA9 and PWR are critical for HOS15’s nuclear activity.

### Regulation of AGL19 chromatin by a novel chromatin remodeling complex

Recent reports have shown that HOS15-HDA9-PWR is a co-repressor complex which modulates histone acetylation status thereby regulating physiological processes including senescence (Mayer et al., 2019; Zareen et al., 2022). Consistent with previous report, acetylated H3 was accumulated abundantly in *hos15*, *hda9* and *pwr* single, double, and triple mutants. In addition to this, we have also found that HOS15-HDA9-PWR complex regulates flowering time through regulation of *AGL19* chromatin (**Figure 5**). Using chromatin immunoprecipitation assay, we found that *AGL19* chromatin was hyperacetylated in loss of function mutants of HOS15-HDA9-PWR complex (**Figure 5**), highlighting the importance of this complex in floral transition *via AGL19* regulation. In the WT plants, the HOS15-PWR-HDA9 co-repressor complex negatively regulated the expression of *AGL19* by promoting histone deacetylation at the *AGL19* promoter, which compacts chromatin. In the absence of PWR and HDA9, the HOS15 protein was unstable in the nucleus. The removal of either one of the components of the repressor complex prevented histone deacetylation, thereby relaxing *AGL19* chromatin, the increase in the expression of *AGL19* and accelerating the flowering time (**Figure 6**). This study revealed that HOS15 together with HDA9 and PWR control flowering time by repressing *AGL19* through increased histone deacetylation.

## Data availability statement

The original contributions presented in the study are included in the article/supplementary material. Further inquiries can be directed to the corresponding author.

## Author contributions

CL, KP, AA, and D-JY conceived and designed the research. D-JY supervised the experiments. CL, KP, MJ, and SR performed the experiments. MS, JP, HK, ZB, SZ, MB, JC and Z-YX provided technical assistance and advice to CL and KP CJL, KSP, AA, JMP, and D-JY wrote the manuscrip. All authors contributed to the article and approved the submitted version.
