# Supplementary material for: Negative regulation of floral transition in Arabidopsis by HOS15-PWR-HDA9 complex
Source: Front Plant Sci. 2023 Jan 6;13:1105988. doi: 10.3389/fpls.2022.1105988 (PMC9853073; doi:10.3389/fpls.2022.1105988)
Supplement: Supplementary file 1 [file DataSheet_1.docx]

**Supplementary Data.**


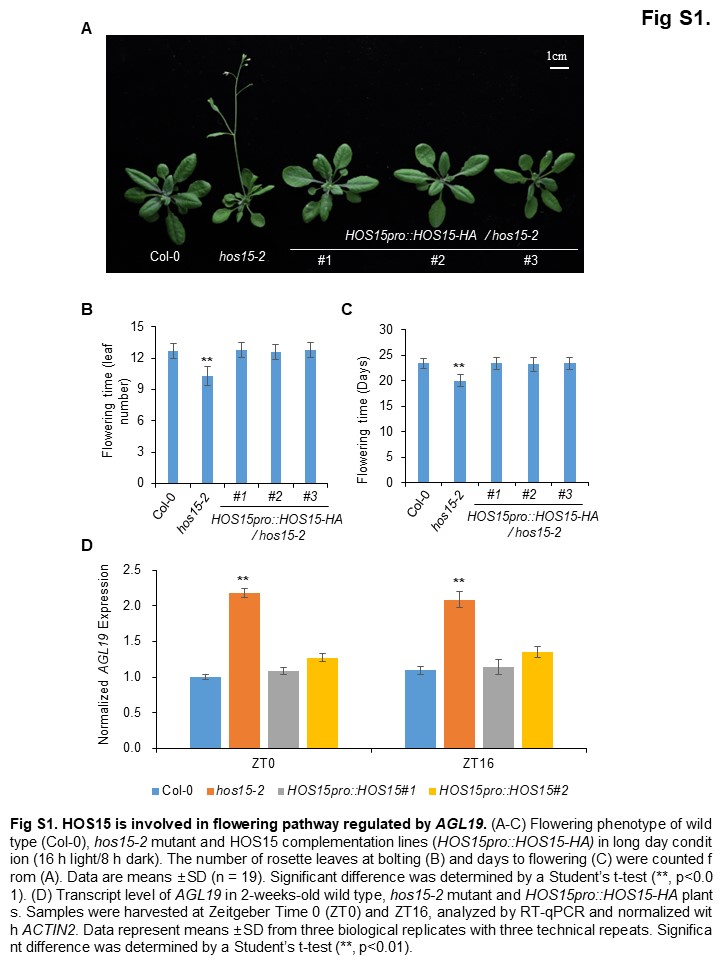


**Fig S1. HOS15 is involved in flowering pathway regulated by *AGL19*.** (A-C) Flowering phenotype of wild type (Col-0), *hos15-2* mutant and HOS15 complementation lines (*HOS15pro::HOS15-HA)* in long day condition (16 h light/8 h dark). The number of rosette leaves at bolting (B) and days to flowering (C) were counted from (A). Data are means ±SD (n = 19). Significant difference was determined by a Student’s t-test (**, p<0.01). (D) Transcript level of *AGL19* in 2-weeks-old wild type, *hos15-2* mutant and *HOS15pro::HOS15-HA* plants. Samples were harvested at Zeitgeber Time 0 (ZT0) and ZT16, analyzed by RT-qPCR and normalized with *ACTIN2*. Data represent means ±SD from three biological replicates with three technical repeats. Significant difference was determined by a Student’s t-test (**, p<0.01).


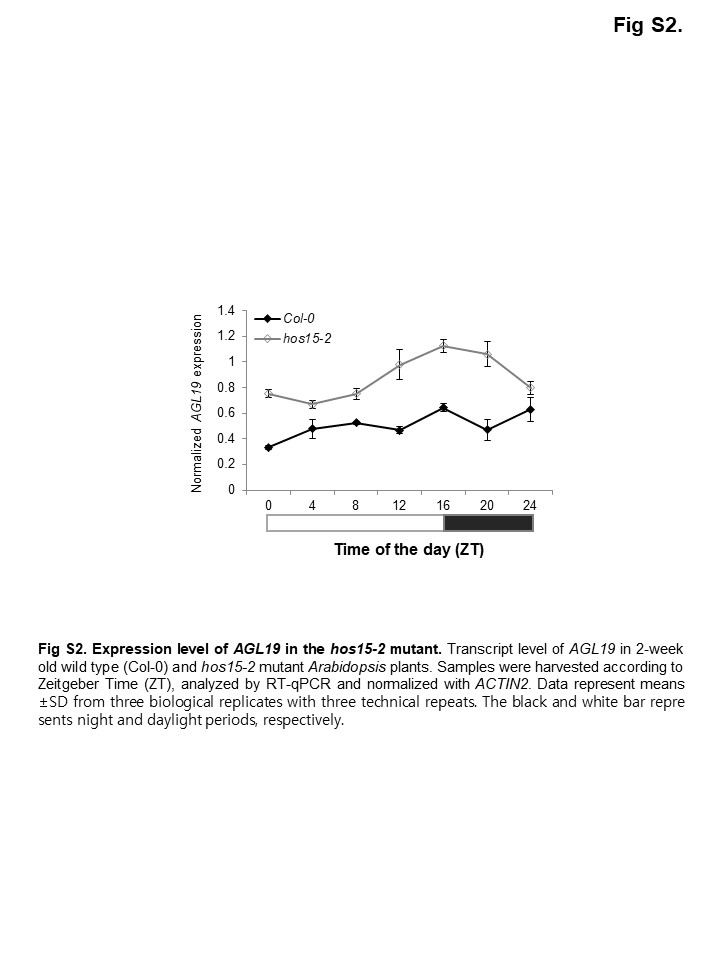


**Fig S2. Expression level of *AGL19* in the *hos15-2* mutant.** Transcript level of *AGL19* in 2-week old wild type (Col-0) and *hos15-2* mutant *Arabidopsis* plants. Samples were harvested according to Zeitgeber Time (ZT), analyzed by RT-qPCR and normalized with *ACTIN2*. Data represent means ±SD from three biological replicates with three technical repeats. The black and white bar represents night and daylight periods, respectively.


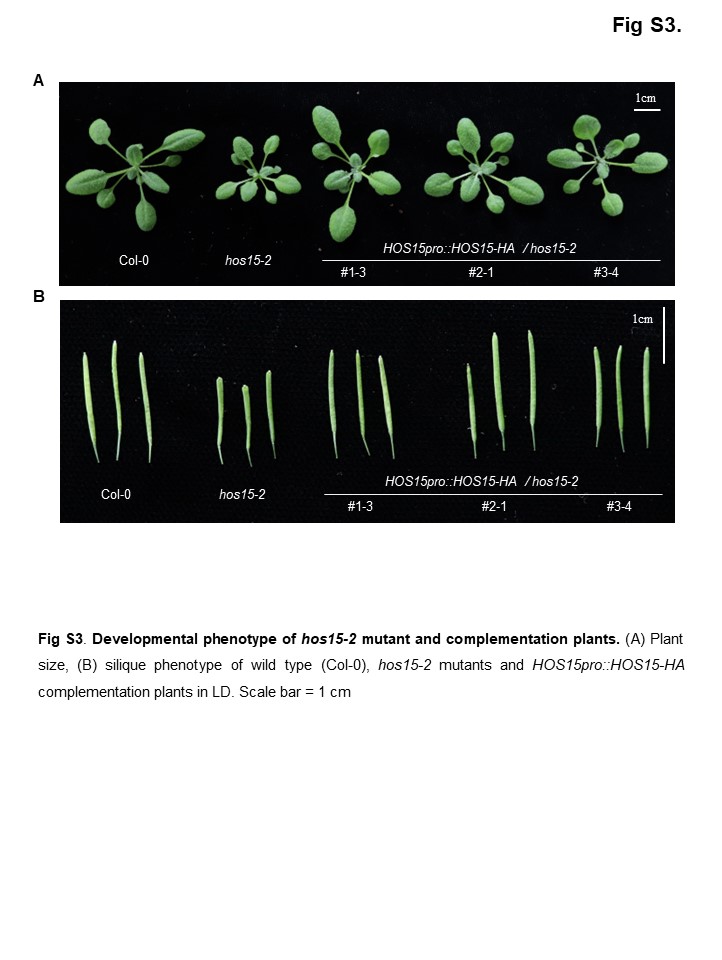


**Fig S3. Developmental phenotype of *hos15-2* mutant and complementation lines.** Plant size (A) and silique phenotype (B) of wild type (Col-0), *hos15-2* mutants and *HOS15pro::HOS15-HA* complementation plants in LD. Scale bar = 1 cm


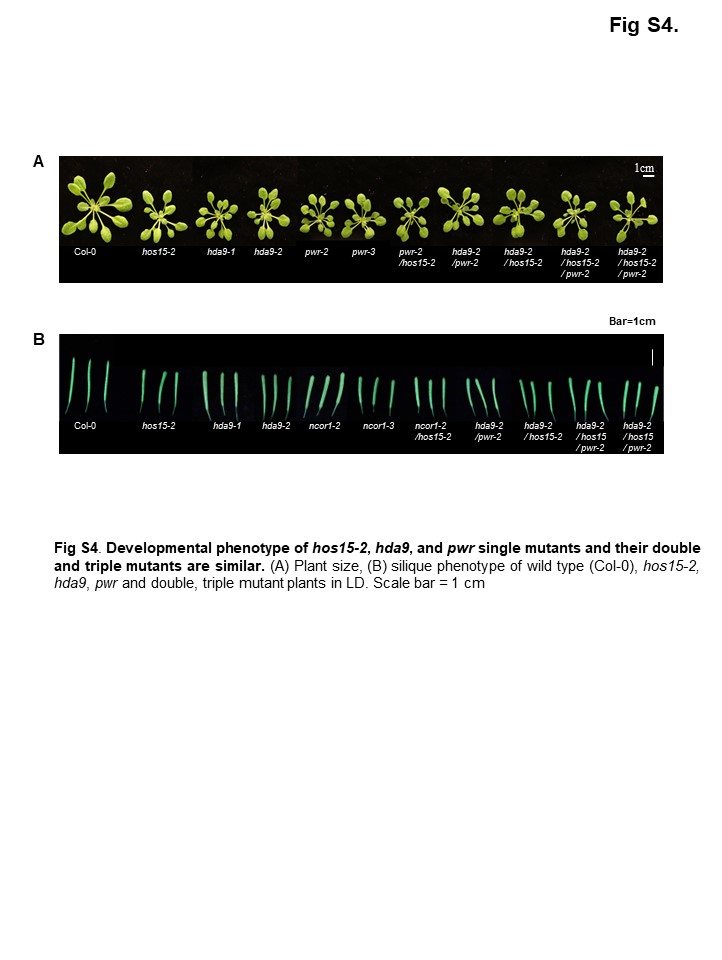


**Fig S4. Developmental phenotypes of *hos15-2*, *hda9*, and *pwr* single, double and triple mutants.** Plant size (A), and silique phenotype (B) of wild type (Col-0), *hos15-2, hda9*, *pwr* and double, triple mutant plants in LD. Scale bar = 1 cm


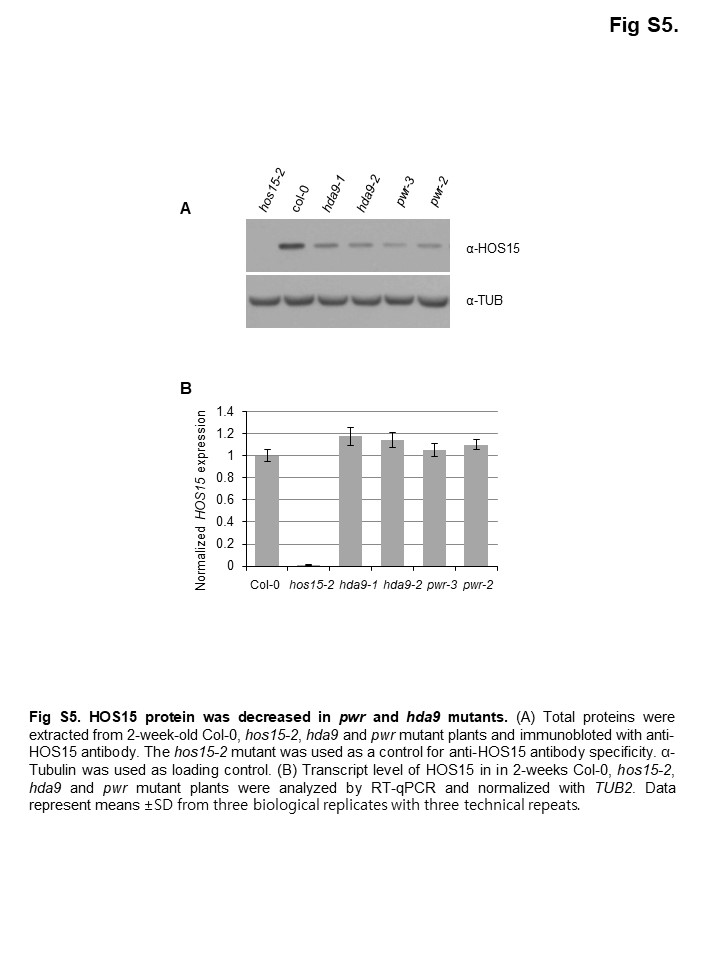


**Fig S5. Reduced HOS15 protein abundance in *pwr* and *hda9* mutants.** (A) Total proteins were extracted from 2-week-old Col-0, *hos15-2*, *hda9* and *pwr* mutant plants and immunobloted with anti-HOS15 antibody. The *hos15-2* mutant was used as a control for HOS15 antibody specificity. α-Tubulin was used as loading control. (B) Transcript level of HOS15 in in 2-weeks Col-0, *hos15-2*, *hda9* and *pwr* mutant plants were analyzed by RT-qPCR and normalized with *TUB2*. Data represent means ±SD from three biological replicates with three technical repeats.


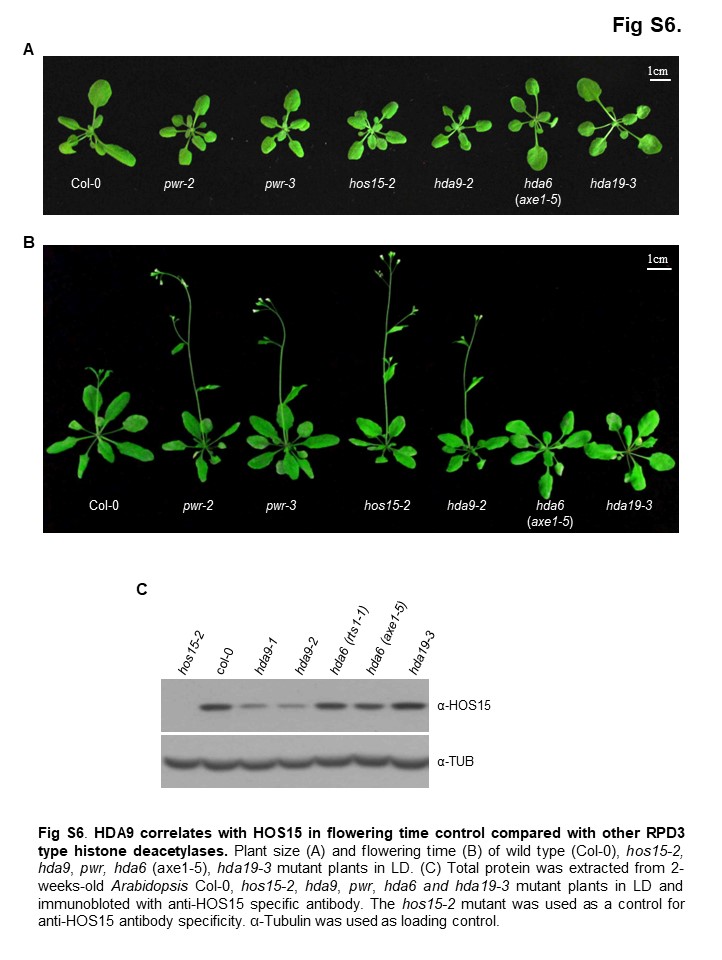


**Fig S6. HDA9 correlates with HOS15 in flowering time control compared with other RPD3 type histone deacetylases.** Plant size (A) and flowering time (B) of wild type (Col-0), *hos15-2, hda9*, *pwr,* *hda6* (axe1-5), *hda19-3* mutant plants in LD. (C) Total protein was extracted from 2-weeks-old *Arabidopsis* Col-0, *hos15-2*, *hda9*, *pwr*, *hda6 and* *hda19-3* mutant plants in LD and immunobloted with anti-HOS15 specific antibody. The *hos15-2* mutant was used as a control for anti-HOS15 antibody specificity. α-Tubulin was used as loading control.


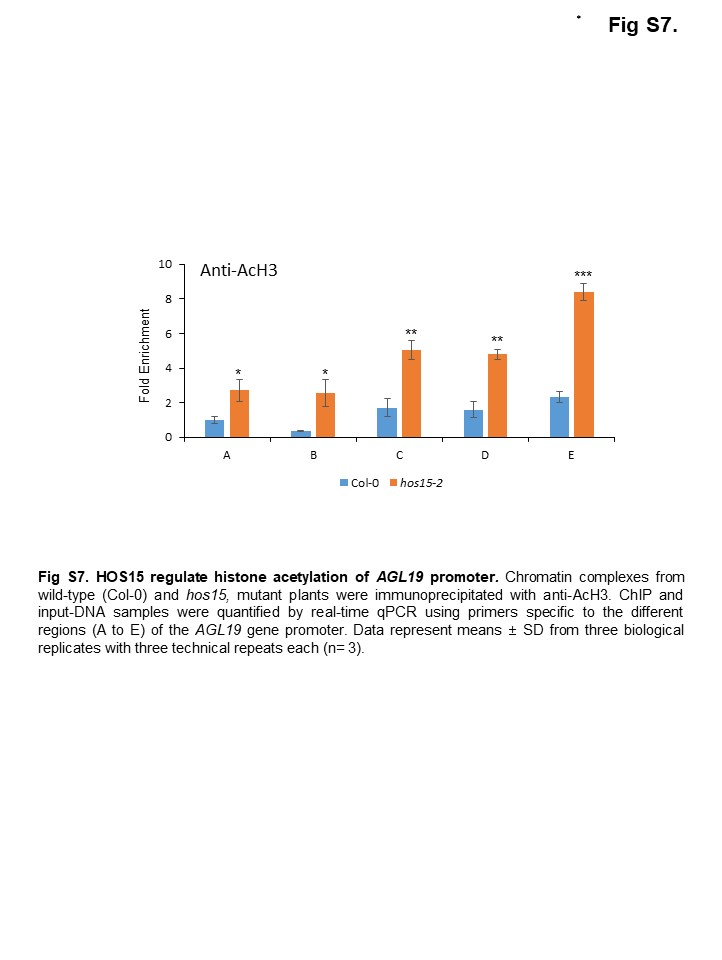


**Fig S7. HOS15 regulate histone acetylation of *AGL19* promoter*.*** Chromatin complexes from wild-type (Col-0) and *hos15,* mutant plants were immunoprecipitated with anti-AcH3. ChIP and input-DNA samples were quantified by real-time qPCR using primers specific to the different regions (A to E) of the *AGL19* gene promoter. Data represent means ± SD from three biological replicates with three technical repeats each (n= 3). Significant difference was determined by a Student’s t-test (*p<0.05, **p<0.01, ***p<0.001,).

Supplemental Table S1. List of primers used in this study

| **Primers** | **Sequences (5' to 3')** | **Purposes** |
| --- | --- | --- |
| AGL19 qRT F | gcagaggagaggaatctcgtta | qRT-PCR |
| AGL19 qRT R | aggtccaatgaacaaaccagtc |  |
| ACTIN2 qRT F | tgggatgaaccagaaggatg |  |
| ACTIN2 qRT R | aagaatacctctcttggattgtgc |  |
| TUB qRT F | tggcatcaactttcattgga |  |
| TUB qRT R | atgttgctctccgcttctgt |  |
| AGL19 AF | ccattgatagattttggatattagataa | ChIP |
| AGL19 AR | caggtgtcgcacgctaggagaggaccaca |  |
| AGL19 BF | gttactgttttatttgtgcgaaggt |  |
| AGL19 BR | ttccacagaagaagcagaactttat |  |
| AGL19 CF | gtatccatttttgtgtcgaagtctttt |  |
| AGL19 CR | tcggacaaaataagtagttaggacacac |  |
| AGL19 DF | ctatccgtagccataagagaaaatg |  |
| AGL19 DR | aagccctagatttatgatgaaggag |  |
| AGL19 EF | tttctttctttctctcccctccttcat |  |
| AGL19 ER | atctatcttctataagtgagtggagagt |  |
| UBQ10 Chip F | ttgccaattttcagctccac |  |
| UBQ10 Chip R | tgactcgtcgacaaccacaa |  |
